# *Ehrlichia chaffeensis* in Child, Venezuela

**DOI:** 10.3201/eid1403.061304

**Published:** 2008-03

**Authors:** María C. Martínez, Clara N. Gutiérrez, Franklin Monger, Johanny Ruiz, Akemys Watts, Victor M. Mijares, María G. Rojas, Francisco J. Triana-Alonso

**Affiliations:** * University of Carabobo, Aragua, Venezuela; † Biomedical Research Institute, Maracay, Venezuela; ‡ Institute for Advanced Studies, Caracas, Venezuela

**Keywords:** Ehrlichia, ehrlichiosis, human monocytic ehrlichiosis, monocytic ehrlichiosis, Ehrlichia chaffeensis, family Anaplasmataceae, tick exposure, letter

**To the Editor:** Human monocytic ehrlichiosis is a tick-borne infectious disease caused by *Ehrlichia chaffeensis* (*1*). Serologic studies have indicated *E. chaffeensis* infection in Latin American countries: Venezuela (*2*), Mexico (*3*), Argentina (*4*), Chile (*5*), and Brazil (*6*). However, no molecular evidence for *E. chaffeensis* has been reported.

In December 2001, a 9-year-old boy was admitted to a hospital in Carabobo, Venezuela, after 3 days of fever (39°C–41°C), malaise, anorexia, headache, abdominal pain, and cutaneous tick-bite lesions. During the 6 weeks before admission, the patient had been exposed to ticks in a rural area (Cojedes, Venezuela). At the time of physical examination, the patient appeared acutely ill with fever (41°C), dehydration, somnolence, conjunctivitis, facial edema, cervical adenomegaly, soft depressible abdomen painful to palpation, and hepatomegaly. Cardiopulmonary examination found regular cardiac sounds with systolic tricuspid murmur and abnormal bilateral respiratory sounds (rhonchi). Skin examination showed multiple tick bites and an erythematous maculopapular rash. Appropriate informed consent was obtained.

Blood values were as follows: leukocytes 6,280 cells/mm^3^ (84% neutrophils, 13% lymphocytes, 2% monocytes, 1% eosinophils), platelets 130,000/μL, hemoglobin 12.5 g/dL, glucose 102 mg/dL, blood urea 28.3 mg/dL, creatinine 0.9 mg/dL, aspartate aminotransferase (AST) 20.4 U/L, alanine aminotransferase (ALT) 54.4 U/L, erythrocyte sedimentation rate (Katz index) 15 mm/h, prothrombin time ratio 1.02, partial thromboplastin time –2.8 s. Radiographs of the thorax showed bilateral infiltrate. Echocardiogram showed minor tricuspid insufficiency. Serologic tests were negative for Epstein-Barr and hepatitis B and positive for cytomegalovirus and hepatitis A viruses. Blood and stool cultures were negative. Blood samples were taken 4 and 35 days after illness onset; buffy-coat smears were stained with Dip Quick (Jorgensen Laboratories, Inc., Loveland, CO, USA), and immunologic and PCR tests were performed. Immunoglobulin (Ig) M against dengue virus was present at days 4 and 35 of illness; IgG against dengue was absent on day 4 and present on day 35. PCR and viral isolation tests for dengue virus were negative. Serologic tests for *E. chaffeensis* (indirect immunofluorescence) were also negative on day 4 and positive (256) on day 35. Detection of *Ehrlichia* species–specific DNA was performed by using nested PCR as described (*7*).

Starting on the first day of hospitalization, the patient was treated with doxycycline (14 days) and chloramphenicol (8 days). After 24 hours, malaise, headache, facial edema, and conjunctivitis improved. After 48 hours, fever and rash were gone. After 3 days, his appetite improved; progressively over time, cervical adenomegaly and cutaneous lesions improved. Abdominal pain persisted for 7 days after treatment. Nausea and vomiting started 2 days after admittance; on day 7, vomit was of coffee-ground consistency. All remaining symptoms abated thereafter. The patient had diarrhea during days 3–6 after admittance; hepatomegaly disappeared after 4 days. Ultrasonographic images of the abdomen indicated acute cholecystitis and hepatosplenomegaly; endoscopic examination of the upper digestive tract showed hyperplasia, hyperemia, and linear and pseudomembranous lacerations in the middle and distal thirds of the esophagus (Mallory-Weiss syndrome) and moderate erythema of the stomach. Test results for *Helicobacter pylori* and *Giardia lamblia* were negative.

Laboratory results showed leukopenia and monocytosis on day 5 of illness. Leukocyte count was within reference range thereafter; thrombocytopenia was present until day 7 (99,000/mm^3^). ALT was elevated from day 3 and peaked (481 IU) on day 7. AST levels increased on day 5 and peaked (215 IU) on day 7. Both values decreased progressively to reference levels (after 25 days for ALT and 46 days for AST). Lactic dehydrogenase was elevated for 9 days while erythrocyte count, sedimentation rate, and serum glucose, amylase, urea, creatinine, bilirubin, calcium, sodium, and potassium remained within reference limits. The patient was released after 8 days of hospitalization.

The buffy-coat smear performed 4 days after illness onset showed basophilic intracytoplasmic inclusions inside vacuoles of lymphocytes and monocytes, with typical features of morulae reported for human monocytic ehrlichiosis (Figure). Nested PCR analysis was positive for *E. chaffeensis,* and sequencing of the amplified DNA fully confirmed the 16S rRNA targeted sequence.

**Figure:**
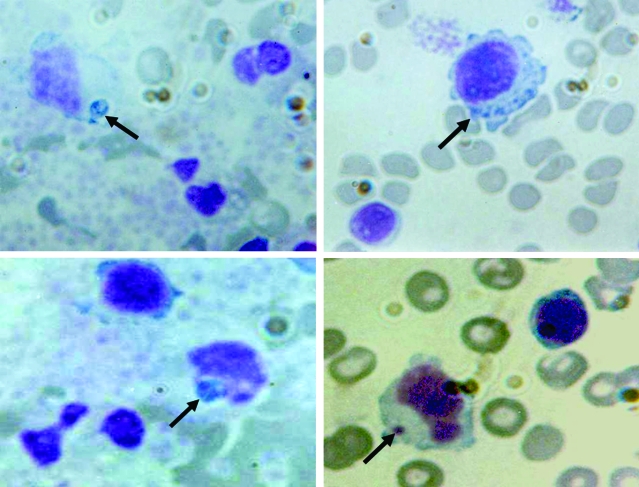
Peripheral blood smears (buffy-coat preparation) showing variable-sized basophilic inclusions (arrows) in mononuclear cells from a 9-year-old boy with human monocytic ehrlichiosis, Carabobo, Venezuela. Dip Quick (Jorgensen Laboratories, Inc., Loveland, CO, USA) staining; magnification ×1,000.

This report provides molecular evidence of *E. chaffeensis* infection in a patient with acute disease in Venezuela. A previous case of human monocytic ehrlichiosis in a 17-month-old girl in Venezuela has been demonstrated serologically (*2*). *E. canis* in an asymptomatic patient in Venezuela has been demonstrated by PCR and culture isolation (*8*) and was recently demonstrated in symptomatic patients (*9*). Excluding the esophageal lesions (Mallory-Weiss syndrome), our case is compatible with cases reported previously (*10*). The clinical manifestations of ehrlichiosis are similar to those of dengue fever and mononucleosis, both common diseases in Venezuela. The positive anti-dengue IgM and the seroconversion of the IgG together with the negative PCR and isolation results suggest a recent, inactive infection with dengue virus.

According to our findings, ehrlichiosis should be a differential diagnosis for febrile patients who have thrombocytopenia, hepatomegaly, and recent exposure to ticks. Although *Amblyomma americanum*, the main known vector of *E. chaffeensis*, has not been reported in Venezuela, *Rhipicephalus sanguineus* and *A.* *cajennense* are abundant in rural areas of Venezuela; their ability to be vectors should be investigated.
